# Current Limitations and Perspectives of Chimeric Antigen Receptor-T-Cells in Acute Myeloid Leukemia

**DOI:** 10.3390/cancers13246157

**Published:** 2021-12-07

**Authors:** Marius Maucher, Micha Srour, Sophia Danhof, Hermann Einsele, Michael Hudecek, Ibrahim Yakoub-Agha

**Affiliations:** 1 Medizinische Klinik und Poliklinik II, Universitätsklinikum Würzburg, Oberdürrbacherstraße 6, 97080 Würzburg, Germany; Maucher_M@ukw.de (M.M.); Danhof_S@ukw.de (S.D.); Einsele_H@ukw.de (H.E.); Hudecek_M@ukw.de (M.H.); 2 Service des Maladies du Sang, CHU de Lille, Université de Lille, 59000 Lille, France; micha.srour@chru-lille.fr; 3 Institute for Translational Research in Inflammation (Infinite), INSERM U1286, CHU de Lille, Université de Lille, 59000 Lille, France

**Keywords:** AML, CAR-T-cell, hematology, gene therapy, adoptive cell therapy

## Abstract

**Simple Summary:**

Acute myeloid leukemia (AML) is the most frequent type of acute leukemia in adults. Allogeneic hematopoietic cell transplantation (allo-HCT) has been the only potentially curative treatment for the majority of patients. The ability of chimeric antigen receptor (CAR)-modified T-cell therapy directed against the CD19 antigen to induce durable remissions in patients with acute lymphoblastic leukemia (ALL) has provided optimism that this novel treatment paradigm can be extrapolated to AML. In this review, we provide an overview of candidate target antigens for CAR-T-cells in AML, an update on recent progress in preclinical and clinical development of investigational CAR-T-cell products, and discuss challenges for the clinical implementation of CAR-T-cell therapy in AML.

**Abstract:**

Adoptive transfer of gene-engineered chimeric antigen receptor (CAR)-T-cells has emerged as a powerful immunotherapy for combating hematologic cancers. Several target antigens that are prevalently expressed on AML cells have undergone evaluation in preclinical CAR-T-cell testing. Attributes of an ‘ideal’ target antigen for CAR-T-cell therapy in AML include high-level expression on leukemic blasts and leukemic stem cells (LSCs), and absence on healthy tissues, normal hematopoietic stem and progenitor cells (HSPCs). In contrast to other blood cancer types, where CAR-T therapies are being similarly studied, only a rather small number of AML patients has received CAR-T-cell treatment in clinical trials, resulting in limited clinical experience for this therapeutic approach in AML. For curative AML treatment, abrogation of bulk blasts and LSCs is mandatory with the need for hematopoietic recovery after CAR-T administration. Herein, we provide a critical review of the current pipeline of candidate target antigens and corresponding CAR-T-cell products in AML, assess challenges for clinical translation and implementation in routine clinical practice, as well as perspectives for overcoming them.

## 1. Introduction

Acute myeloid leukemia (AML) is a clonal proliferative neoplasm usually characterized by bone marrow, blood, and other tissue infiltration. Current AML cure rates range from 35% to 40% and 5% to 15% in patients over 60 years and up to and including 60 years of age, respectively [1]. The prognosis depends on the molecular and cytogenetic features approved by the 2016 revision of the WHO classification of myeloid neoplasm and acute leukemia [2] and the ELN recommendation [3]. Treatment is determined according to the risk stratification based on the WHO classification. Intensive induction therapy remains the backbone of therapy in younger patients, while hypomethylating agents are recommended in older patients [4]. For the last three decades, allogeneic hematopoietic cell transplantation (allo-HCT) has been the first immune-based therapy and only potentially curative approach widely used in intermediate and high-risk AML. Despite considerable improvement in the conditioning regimen, HLA donor selection, and stem cell sourcing, non-relapse mortality remains a major concern. Because allo-HCT is restricted to a subset of relatively young patients with a low comorbidity index and good performance status [5,6,7], new tolerable and effective therapeutic approaches are needed. 

Over the past two decades, AML and other hematological malignancies have become targets for the developing field of immune therapy, such as antibody-based therapeutics, dendritic cell vaccines, TCR-T-cell therapy, and gene-engineered chimeric antigen receptor (CAR)-T-cells, coupled with conventional chemotherapy and molecular targeted therapy [8,9,10,11]. Following the discovery of immune inhibitory mechanisms and cancer-related antigens on leukemic cells, immune approaches are evolving constantly. 

## 2. Emerging CAR-T-Cell Therapy in AML

Adoptive transfer of gene-engineered CAR-T-cells has emerged as a powerful arm of immunotherapy to combat hematologic cancers [12,13,14]. These artificial receptors are genetic hybrids of antibody and T-cell receptor (TCR) domains that are composed of an extracellular binding unit, typically a single-chain variable fragment (scFv) at the N-terminus connected to spacer or hinge domains, a transmembrane domain, and an intracellular signal module at the C-terminus [12,15]. The principle behind CARs is to elicit a cellular anti-tumor response through an artificial receptor that acquires specificity for distinct tumor-associated antigens (TAAs). We currently distinguish between at least four generations of CARs. First-generation CARs only have the CD3ζ domain as intracellular compound, whereas second-generation CARs are built from the CD3ζ and a co-stimulatory domain such as 4-1BB or CD28 [16,17]. Adding a co-stimulatory domain improved efficacy, expansion, and persistence of CAR-T-cells [18,19]. Third-generation CARs carry two co-stimulatory domains in the same receptor, e.g., 4-1BB and CD28, while fourth-generation CAR-T-cells consist of one antigen-specific receptor with the addition of other transgenes that enhance anti-tumor function [20,21,22]. All CAR-engineered cell products are designed to eliminate tumor cells upon cell surface target antigen recognition [12]. In the case of CAR-T-cells, an anti-tumor memory that will prevent relapse of antigen-positive cancer cells may be established [23].

The growing evidence of T-cell anti-leukemic responses derived from healthy donors in the context of allo-HCT has resulted in leukemic and lymphoid malignancies becoming a focus of advanced research into T-cell based immunotherapy [24]. This research has rapidly established CD19 as a key target antigen for B-cell lymphomas and leukemias. This has led to the drug approval of blinatumomab and other bispecific biologicals engaging CD19 and T-cells, as well as the approval of autologous CD19-directed CAR-T-cell products [25,26]. Four CAR-T-cell products targeting CD19 and one targeting B-cell maturation antigen (BCMA) have been approved by the FDA: tisagenlecleucel (Kymriah^®^, Novartis, Basel, Switzerland), axicabtagene ciloleucel (Yescarta^®^, Gilead, Foster City, CA, USA), brexucabtagene autoleucel (Tecartus^®^, Gilead), lisocabtagene maraleucel (Breyanzi^®^, Bristol-Myers Squibb, New York, NY, USA), and idecabtagene vicleucel (Abecma^®^, Bristol-Myers Squibb) [27,28,29].

A customized ‘living drug’, autologous CAR-T-cell production is a sophisticated process demanding careful and precise manufacturing and logistics. As a first step, leukocytes of cancer patients are obtained by leukapheresis. Subsequent isolation of T-cells is performed depending on the specific T-cell product and the manufacturer [30,31]. Enriched T-cells are activated, typically via anti-CD3/CD28 magnetic beads or antibody mixtures, and transduced with retroviral or lentiviral vectors to stably express the CAR [12,15,32] and expanded with cytokine supplementation. All commercial CAR-T-cells are delivered to the treatment center as frozen products, thawed and infused into the patients as a single dose. Prior lymphodepletion is generally foreseen in treatment protocols as CAR-T-cell expansion after injection is markedly promoted [33].

Ongoing research is seeking to extend CAR-T treatment to other cancer entities, such as solid tumors, while rendering CAR-T-cell manufacture safer and more time-and cost-efficient. Because lenti- and retroviral vectors are expensive to produce and demand careful preparation and application, virus-free gene transfer methods have clear advantages [34,35]. Transposon-transposase gene delivery platforms, e.g., the Sleeping Beauty transposon system, are increasingly popular in preclinical CAR-T manufacturing and have been successfully tested in clinical trials (NCT04499339 [33,34,36]). Targeted genome-editing tools, first and foremost CRISPR-Cas9, offer additional possibilities for advanced CAR-T-cell engineering [37,38,39].

One of the main challenges in clinical CAR-T-cell usage is the appearance of cytokine release syndrome (CRS) and immune effector cell-associated neurotoxicity syndrome (ICANS) [12,23,40]. Recent therapeutic advances have been made by interfering with the CRS ‘lead’ cytokines IL-1, IL-6 and GM-CSF [41,42,43], in developing so-called STOP-CARs [44], and by successful administration of the tyrosine kinase inhibitor Dasatinib as a pharmacological CAR-T-cell on/off-switch [45].

## 3. AML Target Antigens under Investigation

**Figure 1:**
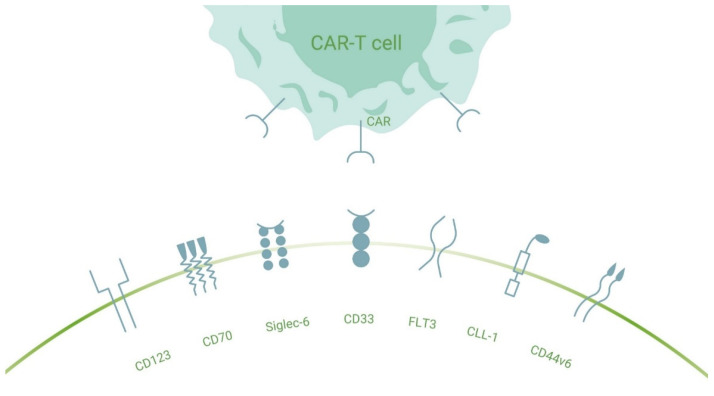
Selection of investigated surface antigen molecules for CAR-T-cell therapy in AML. Depicted membrane proteins on AML blasts or LSCs are recognized by their matching chimeric antigen receptors on gene-engineered T-cells. Abbreviations: CD123: the alpha subunit of the interleukin IL-3 receptor; CD70: the physiological ligand of CD27; CD33: a member of sialic acid-binding immunoglobulin-like lectins (Siglecs); Siglec-6: a member of CD33 (Siglec-3)-related protein superfamily; FLT3: FMS-like tyrosine kinase 3; CLL-1: C-type lectin like molecule-1; CD44v6: a ubiquitous glycoprotein that enables cell adhesion and cell-cell contacts.

The development of CAR-T-cell therapy in AML depends on the identification of suitable target antigens. Though several candidate antigens have been evaluated in preclinical experiments, few of them have been found to satisfy all attributes of a ‘perfect’ AML antigen, i.e., high-level expression on leukemic blasts and leukemic stem cells (LSCs) and absence on healthy normal hematopoietic stem and progenitor cells. In addition, the use of different cut-off and threshold values for flow cytometric analyses results in a controversy over when to define AML cells as target antigen positive [46,47]. In contrast to other blood cancer types where CAR-T-cell therapies are being studied, only a small number of AML patients has received CAR-T treatment in clinical trials, resulting in limited clinical experience for this therapeutic approach in AML (Table 1). In the following sections, we give an overview of AML target antigens under current investigation (Figure 1).

### 3.1. Lewis Y Antigen

The first antigen that was targeted with CAR-T-cells in AML was the Lewis Y antigen, as reported by Ritchie et al. in 2013 [48]. Though the patient with the deepest partial remission relapsed, this study provided proof-of-concept and spurred the quest for additional targets and improved CAR-T-cell products in AML.

### 3.2. CD123

CD123 is the alpha subunit of the interleukin IL-3 receptor, enabling proliferation and survival of AML cells by high-affinity stem cell factor (SCF) binding. Because it is a receptor of normal hematopoietic lineage formation and renewal, complete abrogation of CD123 positive cells is undesirable [49]. Almost every AML patient counts positive for this cell surface molecule with sometimes even higher expression in LSCs than in blasts [46,50]. Several groups developed CD123-specific CAR-T-cells and showed efficacy in in vivo models, including against AML blasts with relatively low CD123 expression [51,52,53,54]. These promising outcomes are countered by reports of in vitro toxicity screens utilizing colony forming unit assays that demonstrate significantly hindered differentiation of healthy hematopoietic stem cells when co-cultured with CD123-specific CAR-T-cells [51,55]. Similarly, CD123-specific scFvs inhibited hematopoiesis in preclinical in vivo models [56].

CD123 CAR-T-cells have been administered in completed and ongoing clinical trials (Table 1). Despite severe toxicity observed in most study participants, complete remission was achieved in at least three patients in two studies [57,58,59]. Though CD123 may be an efficient target for CAR-T-cell therapy in AML, enhanced technologies such as logic gating and dual-antigen targeting are desirable to reduce toxicity and increase tolerability.

### 3.3. CD33

CD33 is a member of the sialic acid-binding immunoglobulin-like lectin (Siglec) superfamily. It is primarily expressed on myeloid cell surfaces [60]. Although monocytes and mature granulocytes show the highest expression, CD33 is detectable on normal hematopoietic stem and progenitor cells (HSPCs) [46,61,62], rendering CD33-directed CAR-T-cells, similar to CD123, toxic to healthy bone marrow cells. It is important to note that CD33 is prevalent on the surface of AML cells in greater than 96% of adult patients, with higher expression levels on blasts compared to LSCs [46,63]. While complete tumor eradication remained difficult to achieve, including in animal models [61,64], preclinical results with CD33-specific CAR-T-cells targeting AML cell lines and primary patient samples raised hope for successful clinical translation [65,66,67,68]. Though the first in-human study proved transient reduction of CD33-positive blasts in one patient and a subsequent clinical trial demonstrated complete remission of one study participant, severe but reversible adverse events were reported in one patient, including induced rigorous chills and fevers, drastic fluctuations of preexisting pancytopenia, elevated serum cytokine levels, i.e., interleukin (IL)-6, IL-8, tumor necrosis factor-α, and interferon-γ; slight transient hyperbilirubinemia within two weeks, a subsequent intermittent moderate fever, and reversed fluctuation of the pancytopenia [69]. In contrast, CD33-CAR NK-92 cells have been safely infused in three AML patients eliciting short response in two patients [70]. Considering the ablation of healthy HSPCs, CD33- and CD123- directed CAR-T therapy is therefore most likely best implemented as a bridging therapy between primary chemotherapy and allogeneic hematopoietic cell transplantation (allo-HCT).

### 3.4. CD44v6

CD44 is a ubiquitous glycoprotein that enables cell adhesion and cell-cell contacts through which it seems to play a role in cancer initiation [71]. Because the isoform variant 6 (CD44v6) is expressed in 64% of AML cells, but absent in HSPCs [72], HSPCs have been spared from CAR-T-cells that target CD44v6 in preclinical models [73]. The first clinical experience with low doses of CD44v6 CAR-T-cells demonstrated a favorable toxicity profile (personal communication C. Traversari, Table 1). 

### 3.5. FLT3

FMS-like tyrosine kinase 3 (FLT3) is a key player in normal hematopoiesis and is therefore expressed on HSCs, myeloid, and lymphoid immune cells [74,75,76]. Through tumor growth promotion, FLT3 is uniformly present in AML blasts [74,76,77]. In many AML cases, FLT3 is mutated such that its intracellular kinase domain is constitutively active. Most prominent aberrations are internal tandem duplications (ITDs) in juxta membrane domain as well as tyrosine kinase domain mutations [74,78,79]. Specifically targeting FLT3-mutated AML patient cells with FLT3-specific CAR-T-cells should carry a low risk for FLT3 antigen loss as the mutated FLT3 represents an AML ‘driver mutation’ [80]. FLT3 CAR-T-cell efficacy could be enhanced synergistically both in vitro and in vivo by co-administration of crenolanib, a specific type-I-inhibitor that targets the active FLT3 kinase conformation [80,81]. Because normal hematopoiesis is hampered by FLT3 CAR-T therapy, an adoptive therapy with FLT3 CAR-T-cells will most likely require incorporation into a treatment algorithm with subsequent CAR-T-cell depletion and allo-HCT. Under these circumstances, carefully conducted clinical trials are warranted, especially for high-risk FLT-ITD+ AML patients. Three clinical trials have been initiated with CAR-T-cells against FLT3 in AML (NCT05023707, NCT03904069, NCT05017883).

### 3.6. CD70

CD70 is the physiological ligand of CD27. It belongs to the tumor necrosis factor superfamily and occurs as type II transmembrane glycoprotein that can be shed off to soluble ligand form [82,83]. Monocytes and regulatory T-cells show the highest expression of CD70, facilitating expansion of cytolytic, mostly virus-specific CD8+ T-cells. In addition to overexpression in multiple solid tumors, i.e., renal cell carcinoma, CD70 overexpression has been described for various lymphoid and myeloid cancers, including AML. More than 85% of AML patient samples were CD70-positive with both expression on blasts and LSCs [84,85]. A recent pre-clinical study reported that CD70-directed CAR-T-cells are efficient against AML cells, thereby sparing normal HSCs. Virus-specific T-cells were also recognized and eliminated, constituting an on-target off-tumor toxicity to be considered [86]. Because some healthy tissues, e.g., endothelial cells, also express CD70, careful monitoring for on-target off-tumor reactivity is warranted. Human Proteinatlas: https://www.proteinatlas.org/ENSG00000125726-CD70 (accessed on 24 November 2021). 

### 3.7. Siglec-6

Siglec-6 is a member of the CD33 (Siglec-3)-related protein superfamily, resembling CD33 molecule composition and structure [87,88,89]. Because Siglec-6 contains intracellular immunoreceptor tyrosine-based inhibition motifs (ITIMs), it is considered a negative activation regulator in immune cells [87,88,89]. Its expression pattern is highly restricted to a few healthy cell types and tissues, comprising B-cells, mast cells, basophilic granulocytes, and placenta [88,90,91,92]. In malignant cells, Siglec-6 expression has been described for mucosa-associated lymphoid tissue lymphoma, thyroid cancer, CLL, and clonal mast cell diseases [88,93,94,95]. A recent study reported absence of Siglec-6 expression on normal HSPCs and other healthy blood and solid tissue cells, and demonstrated anti-leukemic efficacy of engineered T-cells with a Siglec-6-specific CAR against AML cells in vitro and in vivo [55]. Although Siglec-6 expression varies among AML patients, high expressors are expected to benefit from Siglec-6-directed CAR-T therapy. Because this treatment strategy does not cause HSPC ablation, it may not require subsequent allo-HCT. Clinical studies of this novel gene therapy are highly recommended [55].

### 3.8. CLL-1

C-type lectin like molecule-1 (CLL-1/CLEC12A) is another target candidate for anti-AML CAR-T therapies. As orphan type II transmembrane glycoprotein receptor, its expression has been reported for AML blasts of nearly every pediatric patient and approx. 80% of adult patients [46,63]. In addition, higher expression levels on LSCs than on normal HSCs have been described, with a distinct incidence in childhood AML patients [46,63]. More than 60% of CD33+ AML patient samples were found to be CLL-1 positive [96]. CLL-1 is not exclusively expressed on a subset of early hematopoietic cells, AML blasts, and LSCs, but it is also present on healthy monocytes and other immune cells [97,98]. The first clinical applications of CLL-1-targeting CAR-T-cells led to complete remission in three pediatric patients who underwent allo-HCT afterwards [99]. Further investigations are needed in order to determine if these encouraging outcomes can be achieved in adult AML patients (Table 1).

### 3.9. Other Target Antigens

Multiple additional antigens have been tested preclinically or in a clinical setting in the quest for better CAR-T cell target proteins. Apart from those described above, the following antigens have either been examined for AML or are being investigated: CD7, CD13, CD19, CD34, CD38, CD56, CD117, B7-H3, mesothelin, NKG2D ligands, IL-10 receptor, GM-CSF receptor, ILT3, TIM-3, MUC-1, Lewis Y, and folate receptor β [46,100,101,102,103,104,105,106,107,108,109] (Table 1).

## 4. Challenges and Perspectives for Successful Implementation of CAR-T-Cell Therapy in AML

### 4.1. Considerations for the Choice of Target Antigen

#### 4.1.1. Antigen Selection

In general, stable and homogenous expression of the target antigen on the tumor cell surface is a prerequisite for potent CAR-T-cell reactivity, whereas absence from relevant healthy tissues is required to avoid on-target/off-tumor toxicities [110,111]. The latter is particularly challenging in AML due to lack of specific tumor-restricted antigens that are not simultaneously co-expressed on normal hematopoietic stem cells or myeloid progenitors.

Different tumor-specific neoantigens that arise from AML-specific mutations have been identified, even though AML is a disease with very low mutational burden [112]. Neoantigens are potentially of high tumor specific immunogenicity, e.g., NPM1 [113], IDH1/2 [114], or pml/RAR alpha [115], but are ‘intracellular antigens’ and therefore inaccessible for conventional CAR binding. Based on the four-nucleotide duplication in the oncogene nucleophosmin (NPM1c), a recent study presented a CAR that binds to the NPM1c-HLA-A2 complex, but not HLA-A2 alone or in complex with other peptides [116]. Similarly, prototypic CARs with TCR-like binding to WT1-HLA-A2 complexes have been generated [117]. Though both approaches demonstrated potent preclinical anti-leukemic activity and while it is attractive to include intracellular target antigens for CAR-T-cell therapy, the downregulation or loss of HLA expression, as previously demonstrated in AML [118], is a pitfall of this strategy.

Alternative splicing is yet another mechanism resulting in relatively tumor-restricted isoform variants. Mis-splicing has been demonstrated for various genes in AML, including FLT3, NOTCH2 [119], and CD44 [73].

#### 4.1.2. Modulation of CAR-T-Cell Persistence

Controlling the persistence of CAR-T-cells is another approach for improving CAR-T-cell safety in the absence of an ideal target antigen. The incorporation of safety switches that eliminate CAR-T-cells in case of major toxicities is one such strategy, exemplified by the herpes simplex virus-thymidine kinase (HSV-TK) suicide gene system developed over 25 years ago [120,121]. A more recently developed suicide strategy relies on the inducible caspase 9 (iCasp9) that dimerizes after administration of the small-molecule AP1903, resulting in downstream activation of pro-apoptotic molecules [122]. Multiple clinical trials with CAR-T-cells have incorporated this safety strategy, e.g., NCT03594162, NCT03696784, NCT03721068, yet other one for haploidentical stem cell transplantation [123], clinical use of the iCas9 suicide switch has not been reported in the context of CAR-T-cell therapy to date. A further strategy for targeted CAR-T cell depletion is the co-expression of a truncated EGFR on the T-cell surface. This makes the engineered T-cells targetable with the anti-EGFR monoclonal antibody cetuximab [124]. Though this strategy has been incorporated into clinical protocols, i.e., NCT02028455, NCT04483778, and NCT04499339, a systematic clinical evaluation of the kinetic and extent of CAR-T cell depletion by this strategy is yet to be performed.

In order to prevent long-term persistence of CAR-T-cells without incorporating a suicide mechanism, mRNA electroporation was used to generate CAR-T cells used in a clinical study evaluating CD123-specific CAR-T-cells for the treatment of AML [57]. Though seven patients were to receive repetitive doses of CAR-T-cells, less than 60% of planned doses were successfully manufactured. No anti-leukemic activity was noted, and the study was terminated prematurely. The mRNA-based approach to CAR-T-cell engineering is still an attractive option and ongoing preclinical refinements are justified.

Another strategy to interrupt CAR-T-cell function is the use of soluble adaptor molecules that bind to CAR-T-cells that recognize a peptide motif of the adaptor molecule. Given their typically short half-life, the interruption of adaptor molecule infusion facilitates terminating or mitigating CAR-T-cell induced toxicities [125]. Promising data from an ongoing phase I clinical trial using a targeting module directed against CD123 was recently reported [58]. Further updates are eagerly awaited.

The approach of STOP-CARs is intriguing because it can decrease the activity of CAR T-cells temporarily, as opposed to shutting it down completely. The CAR construct consists of a recognition chain (antigen binding) and a signaling chain (T cell activation). A computationally designed protein in the endodomain of these two chains can dimerize into a functional heterodimer without the need of a dimerizing agent. This chemically disruptable heterodimer (CDH) can be disrupted exclusively when small molecule agents, such as the Bcl-XL inhibitors A1331852 and A1155463, are administered. Once dissociated in their monomeric forms, CAR-T-cell activation upon antigen recognition is blocked. The main challenge is that the development of designer fusion proteins bears the risk of creating immunogenic epitopes [44].

#### 4.1.3. Combination Strategies with Allo-HCT

One on-target/off-tumor toxicity of considerable concern for antigens expressed on AML and hematopoietic stem cells is the eradication of the latter, potentially resulting in severe and persistent myelosuppression. In order to broaden the therapeutic window of CD33-directed immunotherapies, several groups have preclinically evaluated the effects of genetically modifying the allograft by deleting CD33 in HSCs [62,126,127]. In murine and non-human primate models, deletion of CD33 from hematopoietic stem cells did not compromise their hematopoietic capacities [62]. A clinical trial to evaluate this approach is ongoing [128].

#### 4.1.4. Multiantigen Targeting

Targeting more than one antigen with CAR-T-cells in order to reduce the risk of antigen escape can be achieved by infusing either more than one CAR-T-cell product of different specificities, or by infusing one CAR-T-cell product with either bicistronic CARs (two independent CARs co-expressed in the same T cell), or bispecific CARs (two scFv binding domains on the same CAR backbone) [129]. Ma and colleagues clinically evaluated the potential of a bicistronic CAR-T-cell product targeting CLL1 on leukemic stem cells and CD33 on bulk AML cells [130] and observed MRD-negativity in seven out of nine patients prior to six patients receiving subsequent allo-HCT. The toxicity profile was considerable with grade 3 CRS in two patients and neurotoxicity in three patients [131].

Increasing the specificity of CAR-T-cells to the malignant cells while sparing healthy tissue can be achieved by engineering T-cells with suboptimal activation upon binding of one antigen and full activation requiring co-stimulation through binding of a second antigen [132,133]. This strategy was pursued by He et al. with a combined bispecific and split CAR (BissCAR) T-cell system [106] directed against the myeloid antigens CD13 and TIM-3. While CD13 binding in the absence of TIM-3 on hematopoietic stem cells resulted in low T-cell activation, simultaneous binding of CD13 and TIM-3 as co-expressed on leukemic stem cells enabled CD28-mediated co-stimulation and led to full T-cell activation. As a result, leukemic stem cells were effectively eliminated while large parts of normal hematopoiesis were spared. Clinical evaluation of this and other strategies, such as synthetic notch receptors [134,135], will be required to fully elucidate the degree of tumor specificity and T-cell functionality for these next-generation CAR-T-cells in AML.

### 4.2. Considerations Regarding the Immunosuppressive Character of AML and the Tumor Microenvironment

#### 4.2.1. Modulation of Soluble Factors in the AML Microenvironment

AML is a disease with an immunosuppressive nature and AML cells have properties comparable to myeloid derived suppressor cells (MDSCs) [136]. STAT3 overexpression or induction commonly found in AML is related to downstream immunosuppressive mechanisms [137], such as the release of indoleamine 2–3 dioxygenase (IDO) and arginase (ARG), both soluble factors that impair T-cell function and drive T-cell apoptosis [138]. 

In addition, IDO promotes Th2 responses that are thought to be counterproductive for CAR-T-cell efficacy [139]. A preclinical strategy that modulates the IDO levels in the tumor microenvironment is the overexpression of miR-153, a tumor-suppressive miRNA, that downregulates the IDO1 expression and enhances CAR-T-cell cytotoxicity in murine models for colonic cancer [140]. Fludarabine and cyclophosphamide, the two lymphodepleting components of the most commonly used conditioning regimen prior to CAR-T-cell transfer, downregulate IDO expression in lymphoma cells and augments CAR-T-cell efficacy [141]. The relevance of either strategy in AML has not been investigated to date. IDO catalyzes the conversion of tryptophan into immunosuppressive metabolites [142] that inhibit the expansion of CAR-T-cells in response to interleukins such as IL-7 or IL-15. Approaches to attenuate the deleterious effects of IDO include engineering strategies to equip CAR-T-cells with IL-secreting properties [143,144] or combining CAR-T-cell therapy with the tyrosine kinase inhibitor sorafenib that increases IL-15 production in FLT3-ITD+ AML [145].

#### 4.2.2. Modulation of Cellular Components in the AML Microenvironment

Accumulation of bone marrow and peripheral blood MDSCs has been reported in patients with MDS and AML [146]. MDSCs directly suppress T-cell activity [147,148] through different mechanisms [149]. Given that CD33 is also expressed on MDSCs, CAR-T-cells targeting CD33 represent an option for depleting MDSCs [61,67]. Various other strategies to inhibit or eliminate MDSCs have been proposed, including the use of phosphodiesterase-5 inhibitors [150], class I histone deacetylase inhibitors [151], and all-trans retinoic acid [152]. Tregs, another relevant immunosuppressive population, are also increased in blood and bone marrow of patients with AML [153,154]. Given that IDO belongs to the factors that drive differentiation into Tregs [155,156], strategies that reduce IDO levels are attractive also in this regard. Another approach to inhibit Tregs by depriving them of IL-2, stemming from CAR-T-cells, is the generation of CARs deficient in Lck signaling [157]; however, the effects on pro-inflammatory bystander cells remain to be determined. Unlike for other types of cancer, the role of immune checkpoints for pathogenesis and treatment of AML remains unclear [158,159]. The fact that single agent therapy checkpoint inhibitors have not yet shown a clear benefit in published clinical trials [160] could be explained by the generally low mutational burden of AML. Combination strategies with hypomethylating agents are under ongoing clinical investigation, and CAR-T-cell therapy may benefit from combinations with both groups of drugs.

#### 4.2.3. Clonal Heterogeneity and Clonal Evolution of AML

AML is clonally and genetically more heterogenous in comparison to ALL, which may contribute to the low response rates with CAR-T-cells in AML [161,162,163]. Consolidation with allo-HCT should therefore be considered in patients undergoing CAR-T-cell therapy.

#### 4.2.4. Combination Therapies with Approved Anti-Leukemic Drugs

Hypomethylating agents belong to the therapeutic backbone of AML treatment. In vitro treatment of AML blasts with decitabine and 5-azacytidine has been shown to result in a significant increase in NKG2D ligand expression and makes AML cells more susceptible to CD33-targeted antibody dependent cellular cytotoxicity [164]. Decitabine has been shown to increase the anti-leukemic effects of CD123 CAR-T-cells by differential methylation leading to the enrichment of naïve and early memory T-cells [165]. Further, 5-azacytidine sensitizes tumor cells to CAR-T-cell therapy by upregulation of the co-stimulatory OX40 ligand [166]. These and other observations [167,168] prompted clinical testing of decitabine-primed CAR-T-cells in B-cell malignancies (NCT04697940, NCT04553393). Even though further relevant mechanisms have yet to be understood, combination strategies of hypomethylating agents with CAR-T-cells appear attractive in the context of AML and should be evaluated clinically.

Current data demonstrates that venetoclax, a bcl-2 inhibitor recently approved for combination AML therapy in the elderly, increased the anti-leukemic effects of T-cells through enhanced secretion of reactive oxygen species [169]. Anthracyclines, a component of AML treatment for decades, are known to induce immunogenic cell death mediated by dendritic cells that elicit T-cell immunity [170,171]. Indeed, first data combining CAR-T-cells with sub-therapeutic doses of doxorubicin showed improved tumor elimination in a murine model of osteosarcoma [172].

The effect of other anti-leukemic drugs on CAR-T-cell performance is less favorable. Especially cytarabine reduces T-cell viability and cytokine secretion even at low doses [173] and depletes early lineage T-cells that are essential for T-cell expansion [174]. Immunosuppressive drugs commonly used in allo-HCT, such as calcineurin inhibitors [175] or mycophenolic acid [176], also limit T-cell activity and represent a potential obstacle to effective CAR-T-cell therapy in the post-allo-HCT setting. Strategies therefore include the implementation of CAR-T-cell therapy into earlier treatment lines in high-risk patients to ensure minimal exposure of T-cells to deleterious anti-leukemic drugs and the use of allogeneic T-cell sources. The latter comes with the risk of inducing or exacerbating graft-versus-host disease (GvHD) in recipients. Recent advances in gene engineering strategies, such as CRISPR–Cas9 technology [177] or TALEN-mediated approaches [178], have opened new possibilities for off-the-shelf CAR-T-cell products. While allogeneic CAR-T-cells are still in preclinical testing for AML [179], a favorable clinical safety profile has been documented in B-cell malignancies [180]. The use of allogeneic αβ T-cells as a source for CAR-T-cell production may require additional genetic engineering such as the deletion of the endogenous T-cell receptor to prevent GvHD [181] and the deletion of HLA class I/II to prevent rejection by the recipient’s immune system. Alternative T-cell sources without inherent alloreactivity, such as γδ T-cells [182] or NK cells derived from healthy donors, cord blood [183], induced pluripotent stem cells [184], and immortalized cell lines, are therefore under investigation. Given the logistical and financial challenges of autologous CAR-T-cell therapy, the development of universal CAR-T-cell products remains highly relevant in AML. 

## 5. Conclusions

For curative AML treatment, abrogation of bulk blasts and LSCs is mandatory with the necessity of hematopoietic recovery after CAR-T-cell administration. Efforts in development of new CAR-T-cell therapies for AML have resulted in a rich pipeline of candidate target antigens and corresponding CAR-T-cell products. Considering that AML is a heterogenous disease, it is conceivable that single antigens or combinations of antigens will have to be selected for patients to receive customized CAR-T-cell products and increase the likelihood for inducing durable responses. 

## Figures and Tables

**Table 1:** Main current active or recruiting clinical trials with CAR-T-cell therapies for AML.

Status	Interventions	Phase	NCT Number	Locations
Recruiting	FLT3 CAR-T cells	Early Phase 1	NCT04803929	Zhejiang Provincial People’s Hospital, China
Recruiting	CLL-1 CAR-T cells	Phase 1	NCT04219163	Texas Children’s Hospital, Texas, United States
Recruiting	CD123/CLL1 CAR-T cells	Phase 2 Phase 3	NCT03631576	Fujian Medical University Union Hospital, China
Recruiting	CD19 CAR-T cells	Phase 2 Phase 3	NCT04257175	Chaim Sheba Medical Center, Israel
Recruiting	CLL-1, CD33 and/or CD123 CAR-T cells	Phase 1 Phase 2	NCT04010877	Shenzhen Geno-immune Medical Institute, China
Recruiting	CLL1 CAR-T cells	Phase 1 Phase 2	NCT04884984	The First Affiliated Hospital of Soochow University, China
Recruiting	CD38 CAR-T cells	Phase 1 Phase 2	NCT04351022	The First Affiliated Hospital of Soochow University, China
Recruiting	CD33, CD38, CD56, CD123, CD117, CD133, CD34 and/or MUC-1 CAR-T cells	n/a	NCT03473457	Southern Medical University Zhujiang Hospital, China
Recruiting	CD33 CAR-T cells	Phase 1	NCT04835519	Beijing Boren Hospital, China
Recruiting	CD33 CAR-T cells	Phase 1 Phase 2	NCT03971799	Children’s Hospital of Los Angeles, California, United States [and others]
Recruiting	CD123 CAR-T cells	Phase 1	NCT04014881	Union Hospital, Tongji Medical College, Huazhong University of Science and Technology, China
Recruiting	CD19 CAR-T cells	Phase 1 Phase 2	NCT03896854	The First Affiliated Hospital of Soochow University, China
Recruiting	B7-H3 CAR-T cells	n/a	NCT04692948	Anhui Provincial Hospital, China
Recruiting	CD44v6 CAR-T cells	Phase 1 Phase 2	NCT04097301	IRCCS San Raffaele, Italy IRCCS Ospedale Pediatrico Bambino Gesù, Italy
Recruiting	CD123 CAR-T cells	Phase 1	NCT04318678	St. Jude Children’s Hospital, Tennessee, United States
Recruiting	Allogeneic CD123 CAR-T cells	Phase 1	NCT03190278	University of California, San Francisco (UCSF)-Helen Diller Family Comprehensive Cancer Center, California, United States [and others]
Recruiting	CD123 CAR-T cells	Phase 1 Phase 2	NCT04272125	Chongqing University Cancer Hospital, China
Recruiting	Allogeneic CD19 CAR-γδT cells	n/a	NCT04796441	Hebei yanda Ludaopei Hospital, China
Recruiting	CLL1 CAR-T cells	Early Phase 1	NCT04923919	No.212 Daguan Road, Xishan District, China
Recruiting	CD123 CAR-T cells	Phase 1 Phase 2	NCT04265963	920th Hospital of Joint Logistics Support Force, China
Recruiting	CLL-1 CAR-T cells	Phase 1	NCT04789408	Washington University School of Medicine, Missouri, United States [and other]
Recruiting	CD19, CD20, CD22, CD10, CD33, CD38, CD56, CD117, CD123, CD34, or MUC-1 CAR-T cells +/− peptide-specific DCs	Phase 1	NCT03291444	Zhujiang Hospital, Southern Medical University, China
Recruiting	CD123 CAR-T cells (autologous vs allogeneic)	Phase 1	NCT02159495	City of Hope Medical Center, California, United States
Recruiting	CD7 CAR-T cells	Phase 1 Phase 2	NCT04762485	The First Affiliated Hospital of Soochow University, China
Active, not recruiting	CD123 CAR-T cells	Phase 1	NCT03766126	University of Pennsylvania, Pennsylvania, United States
Recruiting	CD123 CAR-T cells	Phase 1	NCT04678336	Children’s Hospital of Philadelphia, Pennsylvania, United States
Recruiting	CD123 UniCAR-T cells	Phase 1	NCT04230265	Universitätsklinikum Ulm, Germany [and others]
Recruiting	CD7 CAR-T cells	Phase 1 Phase 2	NCT04033302	Shenzhen Geno-immune Medical Institute, China
Recruiting	NKG2D Ligands CAR-T cells	Phase 1	NCT04167696	Mayo Clinic Cancer Center, Florida, United States [and others]

## References

[1] Döhner H., Weisdorf D.J., Bloomfield C.D. (2015). Acute Myeloid Leukemia. N. Engl. J. Med..

[2] Arber D.A., Orazi A., Hasserjian R., Thiele J., Borowitz M.J., Le Beau M.M., Bloomfield C.D., Cazzola M., Vardiman J.W. (2016). The 2016 revision to the World Health Organization classification of myeloid neoplasms and acute leukemia. Blood.

[3] Döhner H., Estey E., Grimwade D., Amadori S., Appelbaum F.R., Büchner T., Dombret H., Ebert B.L., Fenaux P., Larson R.A. (2017). Diagnosis and management of AML in adults: 2017 ELN recommendations from an international expert panel. Blood.

[4] Dombret H., Seymour J.F., Butrym A., Wierzbowska A., Selleslag D., Jang J.H., Kumar R., Cavenagh J., Schuh A.C., Candoni A. (2015). International phase 3 study of azacitidine vs conventional care regimens in older patients with newly diagnosed AML with >30% blasts. Blood.

[5] Estey E.H. (2020). Acute myeloid leukemia: 2021 update on risk-stratification and management. Am. J. Hematol..

[6] Kaltoum A.B.O., Sellama N., Hind D., Yaya K., Mouna L., Asma Q. (2020). MDR1 gene polymorphisms and acute myeloid leukemia AML susceptibility in A Moroccan adult population: A case-control study and meta-analysis. Curr. Res. Transl. Med..

[7] Boujmia O.K.A., Nadifi S., Dehbi H., Lamchahab M., Quessar A. (2020). The influence of DNMT3A and DNMT3B gene polymorphisms on acute myeloid leukemia risk in a Moroccan population. Curr. Res. Transl. Med..

[8] Tabata R., Chi S., Yuda J., Minami Y. (2021). Emerging Immunotherapy for Acute Myeloid Leukemia. Int. J. Mol. Sci..

[9] Prommersberger S., Jetani H., Danhof S., Monjezi R., Nerreter T., Beckmann J., Einsele H., Hudecek M. (2018). Novel targets and technologies for CAR-T cells in multiple myeloma and acute myeloid leukemia. Curr. Res. Transl. Med..

[10] Tawara I., Kageyama S., Miyahara Y., Fujiwara H., Nishida T., Akatsuka Y., Ikeda H., Tanimoto K., Terakura S., Murata M. (2017). Safety and persistence of WT1-specific T-cell receptor gene−transduced lymphocytes in patients with AML and MDS. Blood.

[11] Anguille S., Van de Velde A.L., Smits E.L., Van Tendeloo V.F., Juliusson G., Cools N., Nijs G., Stein B., Lion E., Van Driessche A. (2017). Dendritic cell vaccination as postremission treatment to prevent or delay relapse in acute myeloid leukemia. Blood.

[12] Freitag F., Maucher M., Riester Z., Hudecek M. (2020). New targets and technologies for CAR-T cells. Curr. Opin. Oncol..

[13] Gauthier J., Yakoub-Agha I. (2017). Chimeric antigen-receptor T-cell therapy for hematological malignancies and solid tumors: Clinical data to date, current limitations and perspectives. Curr. Res. Transl. Med..

[14] Danylesko I., Chowers G., Shouval R., Besser M.J., Jacoby E., Shimoni A., Nagler A., Avigdor A. (2019). Treatment with anti CD19 chimeric antigen receptor T cells after antibody-based immunotherapy in adults with acute lymphoblastic leukemia. Curr. Res. Transl. Med..

[15] Subklewe M., von Bergwelt-Baildon M., Humpe A. (2019). Chimeric Antigen Receptor T Cells: A Race to Revolutionize Cancer Therapy. Transfus. Med. Hemotherapy.

[16] Brocker T., Karjalainen K. (1995). Signals through T cell receptor-zeta chain alone are insufficient to prime resting T lymphocytes. J. Exp. Med..

[17] Gong M.C., Latouche J.-B., Krause A., Heston W.D., Bander N.H., Sadelain M. (1999). Cancer Patient T Cells Genetically Targeted to Prostate-Specific Membrane Antigen Specifically Lyse Prostate Cancer Cells and Release Cytokines in Response to Prostate-Specific Membrane Antigen. Neoplasia.

[18] Krause A., Guo H.-F., Latouche J.-B., Tan C., Cheung N.-K.V., Sadelain M. (1998). Antigen-dependent CD28 Signaling Selectively Enhances Survival and Proliferation in Genetically Modified Activated Human Primary T Lymphocytes. J. Exp. Med..

[19] Porter D.L., Levine B.L., Kalos M., Bagg A., June C.H. (2011). Chimeric Antigen Receptor–Modified T Cells in Chronic Lymphoid Leukemia. N. Engl. J. Med..

[20] Brentjens R.J., Curran K.J. (2012). Novel cellular therapies for leukemia: CAR-modified T cells targeted to the CD19 antigen. Hematology.

[21] Wang L.-C.S., Lo A., Scholler J., Sun J., Majumdar R.S., Kapoor V., Antzis M., Cotner C., Johnson L.A., Durham A.C. (2013). Targeting Fibroblast Activation Protein in Tumor Stroma with Chimeric Antigen Receptor T Cells Can Inhibit Tumor Growth and Augment Host Immunity without Severe Toxicity. Cancer Immunol. Res..

[22] Scarfò I., Maus M.V. (2017). Current approaches to increase CAR T cell potency in solid tumors: Targeting the tumor microenvironment. J. Immunother. Cancer.

[23] Hartmann J., Schüßler-Lenz M., Bondanza A., Buchholz C.J. (2017). Clinical development of CAR T cells—challenges and opportunities in translating innovative treatment concepts. EMBO Mol. Med..

[24] Kolb H.-J., Parties E.I.A.C.L.W. (1998). Donor Leukocyte Transfusions for Treatment of Leukemic Relapse after Bone Marrow Transplantation. Vox Sang..

[25] Yassine F., Iqbal M., Murthy H., Kharfan-Dabaja M.A., Chavez J.C. (2020). Real world experience of approved chimeric antigen receptor T-cell therapies outside of clinical trials. Curr. Res. Transl. Med..

[26] Przepiorka D., Ko C.-W., Deisseroth A.B., Yancey C.L., Candau-Chacon R., Chiu H.-J., Gehrke B.J., Gomez-Broughton C., Kane R.C., Kirshner S. (2015). FDA Approval: Blinatumomab. Clin. Cancer Res..

[27] Beauvais D., Danhof S., Hayden P.J., Einsele H., Yakoub-Agha I. (2020). Clinical data, limitations and perspectives on chimeric antigen receptor T-cell therapy in multiple myeloma. Curr. Opin. Oncol..

[28] (2021). Approvals Expand Multiple Myeloma Treatment Options. Cancer Discov..

[29] Mullard A. (2021). FDA approves fourth CAR-T cell therapy. Nat. Rev. Drug Discov..

[30] Wang X., Rivière I. (2016). Clinical manufacturing of CAR T cells: Foundation of a promising therapy. Mol. Ther.-Oncolytics.

[31] Levine B.L., Miskin J., Wonnacott K., Keir C. (2017). Global Manufacturing of CAR T Cell Therapy. Mol. Ther. Methods Clin. Dev..

[32] Levine B.L., Bernstein W.B., Connors M., Craighead N., Lindsten T., Thompson C.B., June C. (1997). Effects of CD28 costimulation on long-term proliferation of CD4+ T cells in the absence of exogenous feeder cells. J. Immunol..

[33] Kruger S., Ilmer M., Kobold S., Cadilha B.L., Endres S., Ormanns S., Schuebbe G., Renz B.W., D’Haese J.G., Schloesser H. (2019). Advances in cancer immunotherapy 2019—latest trends. J. Exp. Clin. Cancer Res..

[34] Querques I., Mades A., Zuliani C., Miskey C., Alb M., Grueso E., Machwirth M., Rausch T., Einsele H., Ivics Z. (2019). A highly soluble Sleeping Beauty transposase improves control of gene insertion. Nat. Biotechnol..

[35] Hudecek M., Ivics Z. (2018). Non-viral therapeutic cell engineering with the Sleeping Beauty transposon system. Curr. Opin. Genet. Dev..

[36] Prommersberger S., Reiser M., Beckmann J., Danhof S., Amberger M., Quade-Lyssy P., Einsele H., Hudecek M., Bonig H., Ivics Z. (2021). CARAMBA: A first-in-human clinical trial with SLAMF7 CAR-T cells prepared by virus-free Sleeping Beauty gene transfer to treat multiple myeloma. Gene Ther..

[37] Liu X., Zhao Y. (2018). CRISPR/Cas9 genome editing: Fueling the revolution in cancer immunotherapy. Curr. Res. Transl. Med..

[38] Hudecek M., Izsvák Z., Johnen S., Renner M., Thumann G., Ivics Z. (2017). Going non-viral: The Sleeping Beauty transposon system breaks on through to the clinical side. Crit. Rev. Biochem. Mol. Biol..

[39] Kebriaei P., Singh H., Huls M.H., Figliola M.J., Bassett R., Olivares S., Jena B., Dawson M.J., Kumaresan P.R., Su S. (2016). Phase I trials using Sleeping Beauty to generate CD19-specific CAR T cells. J. Clin. Investig..

[40] Yakoub-Agha I., Chabannon C., Bader P., Basak G.W., Bonig H., Ciceri F., Corbacioglu S., Duarte R.F., Einsele H., Hudecek M. (2019). Management of adults and children undergoing chimeric antigen receptor T-cell therapy: Best practice recommendations of the European Society for Blood and Marrow Transplantation (EBMT) and the Joint Accreditation Committee of ISCT and EBMT (JACIE). Haematologica.

[41] Norelli M., Camisa B., Barbiera G., Falcone L., Purevdorj A., Genua M., Sanvito F., Ponzoni M., Doglioni C., Cristofori P. (2018). Monocyte-derived IL-1 and IL-6 are differentially required for cytokine-release syndrome and neurotoxicity due to CAR T cells. Nat. Med..

[42] Giavridis T., Van Der Stegen S.J.C., Eyquem J., Hamieh M., Piersigilli A., Sadelain M. (2018). CAR T cell–induced cytokine release syndrome is mediated by macrophages and abated by IL-1 blockade. Nat. Med..

[43] Sterner R.M., Sakemura R., Cox M.J., Yang N., Khadka R.H., Forsman C.L., Hansen M.J., Jin F., Ayasoufi K., Hefazi M. (2019). GM-CSF inhibition reduces cytokine release syndrome and neuroinflammation but enhances CAR-T cell function in xenografts. Blood.

[44] Giordano-Attianese G., Gainza P., Gray-Gaillard E., Cribioli E., Shui S., Kim S., Kwak M.-J., Vollers S., Osorio A.D.J.C., Reichenbach P. (2020). A computationally designed chimeric antigen receptor provides a small-molecule safety switch for T-cell therapy. Nat. Biotechnol..

[45] Mestermann K., Giavridis T., Weber J., Rydzek J., Frenz S., Nerreter T., Mades A., Sadelain M., Einsele H., Hudecek M. (2019). The tyrosine kinase inhibitor dasatinib acts as a pharmacologic on/off switch for CAR T cells. Sci. Transl. Med..

[46] Haubner S., Perna F., Köhnke T., Schmidt C., Berman S., Augsberger C., Schnorfeil F.M., Krupka C., Lichtenegger F.S., Liu X. (2018). Coexpression profile of leukemic stem cell markers for combinatorial targeted therapy in AML. Leukemia.

[47] Perna F., Berman S.H., Soni R.K., Mansilla-Soto J., Eyquem J., Hamieh M., Hendrickson R.C., Brennan C., Sadelain M. (2017). Integrating Proteomics and Transcriptomics for Systematic Combinatorial Chimeric Antigen Receptor Therapy of AML. Cancer Cell.

[48] Ritchie D.S., Neeson P.J., Khot A., Peinert S., Tai T., Tainton K., Chen K., Shin M., Wall D.M., Hönemann D. (2013). Persistence and Efficacy of Second Generation CAR T Cell Against the LeY Antigen in Acute Myeloid Leukemia. Mol. Ther..

[49] Testa U., Pelosi E., Frankel A. (2014). CD 123 is a membrane biomarker and a therapeutic target in hematologic malignancies. Biomark. Res..

[50] Jiang G., Atenafu E.G., Capo-Chichi J., Minden M.D., Chang H. (2019). Prognostic relevance of CD123 expression in adult AML with normal karyotype. Br. J. Haematol..

[51] Mardiros A., Dos Santos C., McDonald T., Brown C.E., Wang X., Budde L.E., Hoffman L., Aguilar B., Chang W.-C., Bretzlaff W. (2013). T cells expressing CD123-specific chimeric antigen receptors exhibit specific cytolytic effector functions and antitumor effects against human acute myeloid leukemia. Blood.

[52] Riberdy J.M., Zhou S., Zheng F., Kim Y.-I., Moore J., Vaidya A., Throm R.E., Sykes A., Sahr N., Bonifant C.L. (2020). The Art and Science of Selecting a CD123-Specific Chimeric Antigen Receptor for Clinical Testing. Mol. Ther.-Methods Clin. Dev..

[53] Qin H., Edwards J.P., Zaritskaya L., Gupta A., Mu C.J., Fry T.J., Hilbert D.M., LaFleur D.W. (2019). Chimeric Antigen Receptors Incorporating D Domains Targeting CD123 Direct Potent Mono- and Bi-specific Antitumor Activity of T Cells. Mol. Ther..

[54] Arcangeli S., Rotiroti M.C., Bardelli M., Simonelli L., Magnani C.F., Biondi A., Biagi E., Tettamanti S., Varani L. (2017). Balance of Anti-CD123 Chimeric Antigen Receptor Binding Affinity and Density for the Targeting of Acute Myeloid Leukemia. Mol. Ther..

[55] Jetani H., Navarro-Bailón A., Maucher M., Frenz S., Verbruggen C., Yeguas A., Vidriales M.B., González M., Saborido J.R., Kraus S. (2021). Siglec-6 is a novel target for CAR T-cell therapy in acute myeloid leukemia. Blood.

[56] Gill S., Tasian S., Ruella M., Shestova O., Li Y., Porter D.L., Carroll M., Danet-Desnoyers G., Scholler J., Grupp S.A. (2014). Preclinical targeting of human acute myeloid leukemia and myeloablation using chimeric antigen receptor–modified T cells. Blood.

[57] Cummins K.D., Frey N., Nelson A.M., Schmidt A., Luger S., Isaacs R.E., Lacey S.F., Hexner E., Melenhorst J.J., June C.H. (2017). Treating Relapsed/Refractory (RR) AML with Biodegradable Anti-CD123 CAR Modified T Cells. Blood.

[58] Wermke M., Kraus S., Ehninger A., Bargou R.C., Goebeler M.-E., Middeke J.M., Kreissig C., von Bonin M., Koedam J., Pehl M. (2021). Proof of concept for a rapidly switchable universal CAR-T platform with UniCAR-T-CD123 in relapsed/refractory AML. Blood.

[59] Yao S., Jianlin C., Yarong L., Botao L., Qinghan W., Hongliang F., Lu Z., Hongmei N., Pin W., Hu C. (2019). Donor-Derived CD123-Targeted CAR T Cell Serves as a RIC Regimen for Haploidentical Transplantation in a Patient With FUS-ERG+ AML. Front. Oncol..

[60] Walter R.B. (2018). Investigational CD33-targeted therapeutics for acute myeloid leukemia. Expert Opin. Investig. Drugs.

[61] Kenderian S., Ruella M., Shestova O., Klichinsky M., Aikawa V., Morrissette J.J.D., Scholler J., Song D., Porter D.L., Carroll M.C. (2015). CD33-specific chimeric antigen receptor T cells exhibit potent preclinical activity against human acute myeloid leukemia. Leukemia.

[62] Kim M.Y., Yu K.-R., Kenderian S.S., Ruella M., Chen S., Shin T.-H., Aljanahi A.A., Schreeder D., Klichinsky M., Shestova O. (2018). Genetic Inactivation of CD33 in Hematopoietic Stem Cells to Enable CAR T Cell Immunotherapy for Acute Myeloid Leukemia. Cell.

[63] Willier S., Rothämel P., Hastreiter M., Wilhelm J., Stenger D., Blaeschke F., Rohlfs M., Kaeuferle T., Schmid I., Albert M.H. (2021). CLEC12A and CD33 coexpression as a preferential target for pediatric AML combinatorial immunotherapy. Blood.

[64] Schneider D., Xiong Y., Hu P., Wu D., Chen W., Ying T., Zhu Z., Dimitrov D.S., Dropulic B., Orentas R.J. (2018). A Unique Human Immunoglobulin Heavy Chain Variable Domain-Only CD33 CAR for the Treatment of Acute Myeloid Leukemia. Front. Oncol..

[65] Marin V., Pizzitola I., Agostoni V., Attianese G.M.P.G., Finney H., Lawson A., Pule M., Rousseau R., Biondi A., Biagi E. (2010). Cytokine-induced killer cells for cell therapy of acute myeloid leukemia: Improvement of their immune activity by expression of CD33-specific chimeric receptors. Haematologica.

[66] Dutour A., Marin V., Pizzitola I., Valsesia-Wittmann S., Lee D., Yvon E., Finney H., Lawson A., Brenner M., Biondi A. (2012). In VitroandIn VivoAntitumor Effect of Anti-CD33 Chimeric Receptor-Expressing EBV-CTL againstCD33+Acute Myeloid Leukemia. Adv. Hematol..

[67] Hear C.O., Heiber J.F., Schubert I., Fey G., Geiger T.L. (2014). Anti-CD33 chimeric antigen receptor targeting of acute myeloid leukemia. Haematologica.

[68] Pizzitola I., Afonso F.D.A., Rouault-Pierre K., Lassailly F., Tettamanti S., Spinelli O., Biondi A., Biagi E., Bonnet D. (2014). Chimeric antigen receptors against CD33/CD123 antigens efficiently target primary acute myeloid leukemia cells in vivo. Leukemia.

[69] Wang Q.-S., Wang Y., Lv H.-Y., Han Q.-W., Fan H., Guo B., Wang L.-L., Han W.-D. (2015). Treatment of CD33-directed Chimeric Antigen Receptor-modified T Cells in One Patient with Relapsed and Refractory Acute Myeloid Leukemia. Mol. Ther..

[70] Tang X., Yang L., Li Z., Nalin A.P., Dai H., Xu T., Yin J., You F., Zhu M., Shen W. (2018). First-in-man clinical trial of CAR NK-92 cells: Safety test of CD33-CAR NK-92 cells in patients with relapsed and refractory acute myeloid leukemia. Am. J. Cancer Res..

[71] Zöller M. (2011). CD44: Can a cancer-initiating cell profit from an abundantly expressed molecule?. Nat. Rev. Cancer.

[72] Neu S., Geiselhart A., Sproll M., Hahn D., Kuci S., Niethammer D., Handgretinger R. (1997). Expression of CD44 isoforms by highly enriched CD34-positive cells in cord blood, bone marrow and leukaphereses. Bone Marrow Transplant..

[73] Casucci M., Nicolis di Robilant B., Falcone L., Camisa B., Norelli M., Genovese P., Gentner B., Gullotta F., Ponzoni M., Bernardi M. (2013). CD44v6-targeted T cells mediate potent antitumor effects against acute myeloid leukemia and multiple myeloma. Blood.

[74] Gilliland D.G., Griffin J.D. (2002). The roles of FLT3 in hematopoiesis and leukemia. Blood.

[75] Kikushige Y., Yoshimoto G., Miyamoto T., Iino T., Mori Y., Iwasaki H., Niiro H., Takenaka K., Nagafuji K., Harada M. (2008). Human Flt3 Is Expressed at the Hematopoietic Stem Cell and the Granulocyte/Macrophage Progenitor Stages to Maintain Cell Survival. J. Immunol..

[76] Rosnet O., Bühring H.J., Marchetto S., Rappold I., Lavagna C., Sainty D., Arnoulet C., Chabannon C., Kanz L., Hannum C. (1996). Human FLT3/FLK2 receptor tyrosine kinase is expressed at the surface of normal and malignant hematopoietic cells. Leukemia.

[77] Carow C.E., Levenstein M., Kaufmann S.H., Chen J., Amin S., Rockwell P., Witte L., Borowitz M.J., Civin C.I., Small D. (1996). Expression of the hematopoietic growth factor receptor FLT3 (STK-1/Flk2) in human leukemias. Blood.

[78] Kindler T., Lipka D.B., Fischer T. (2010). FLT3 as a therapeutic target in AML: Still challenging after all these years. Blood.

[79] Kuchenbauer F., Kern W., Schoch C., Kohlmann A., Hiddemann W., Haferlach T., Schnittger S. (2005). Detailed analysis of FLT3 expression levels in acute myeloid leukemia. Haematologica.

[80] Jetani H., Garcia-Cadenas I., Nerreter T., Thomas S., Rydzek J., Meijide J.B., Bonig H., Herr W., Sierra J., Einsele H. (2018). CAR T-cells targeting FLT3 have potent activity against FLT3−ITD+ AML and act synergistically with the FLT3-inhibitor crenolanib. Leukemia.

[81] Smith C.C., Lasater E.A., Lin K.C., Wang Q., McCreery M.Q., Stewart W.K., Damon L.E., Perl A.E., Jeschke G., Sugita M. (2014). Crenolanib is a selective type I pan-FLT3 inhibitor. Proc. Natl. Acad. Sci. USA.

[82] Wajant H., Wajant H. (2016). Therapeutic targeting of CD70 and CD27. Expert Opin. Ther. Targets.

[83] Al Sayed M.F., Ruckstuhl C.A., Hilmenyuk T., Claus C., Bourquin J.-P., Bornhauser B.C., Radpour R., Riether C., Ochsenbein A.F. (2017). CD70 reverse signaling enhances NK cell function and immunosurveillance in CD27-expressing B-cell malignancies. Blood.

[84] Riether C., Schürch C.M., Bührer E.D., Hinterbrandner M., Huguenin A.-L., Hoepner S., Zlobec I., Pabst T., Radpour R., Ochsenbein A.F. (2016). CD70/CD27 signaling promotes blast stemness and is a viable therapeutic target in acute myeloid leukemia. J. Exp. Med..

[85] Riether C., Pabst T., Höpner S., Bacher U., Hinterbrandner M., Banz Y., Müller R., Manz M.G., Gharib W.H., Francisco D. (2020). Targeting CD70 with cusatuzumab eliminates acute myeloid leukemia stem cells in patients treated with hypomethylating agents. Nat. Med..

[86] Sauer T., Parikh K., Sharma S., Omer B., Sedloev D.N., Chen Q., Angenendt L., Schliemann C., Schmitt M., Müller-Tidow C. (2021). CD70-specific CAR T cells have potent activity against acute myeloid leukemia without HSC toxicity. Blood.

[87] Crocker P.R., Varki A. (2001). Siglecs in the immune system. Immunology.

[88] Patel N., Brinkman-Van der Linden E.C., Altmann S.W., Gish K., Balasubramanian S., Timans J.C., Peterson D., Bell M.P., Bazan J.F., Varki A. (1999). OB-BP1/Siglec-6 a leptin-and sialic acid-binding protein of the immunoglobulin superfamily. J. Biol. Chem..

[89] Nguyen D.H., Ball E.D., Varki A. (2006). Myeloid precursors and acute myeloid leukemia cells express multiple CD33-related Siglecs. Exp. Hematol..

[90] Baskar S., Suschak J.M., Samija I., Srinivasan R., Childs R.W., Pavletic S.Z., Bishop M.R., Rader C. (2009). A human monoclonal antibody drug and target discovery platform for B-cell chronic lymphocytic leukemia based on allogeneic hematopoietic stem cell transplantation and phage display. Blood.

[91] Yokoi H., Myers A., Matsumoto K., Crocker P.R., Saito H., Bochner B.S. (2006). Alteration and acquisition of Siglecs during in vitro maturation of CD34+ progenitors into human mast cells. Allergy.

[92] Yu Y., Blokhuis B.R.J., Diks M.A.P., Keshavarzian A., Garssen J., Redegeld F.A. (2018). Functional Inhibitory Siglec-6 Is Upregulated in Human Colorectal Cancer-Associated Mast Cells. Front. Immunol..

[93] Chng W.J., Remstein E.D., Fonseca R., Bergsagel P.L., Vrana J.A., Kurtin P.J., Dogan A. (2009). Gene expression profiling of pulmonary mucosa-associated lymphoid tissue lymphoma identifies new biologic insights with potential diagnostic and therapeutic applications. Blood.

[94] Andrea R., Teufelberger J.W., Overed-Sayer C., Ekoff1 M., Dahlén B., Gülen T., Nilsson G.P. Siglec-6: A Potential New Biomarker for Clonal Mast Cell Diseases. https://www.google.com/url?sa=t&rct=j&q=&esrc=s&source=web&cd=&cad=rja&uact=8&ved=2ahUKEwiIm8GNiLT0AhXjUOUKHUw4CcEQFnoECAgQAQ&url=http%3A%2F%2Fdownload2.eurordis.org.s3-eu-west-1.amazonaws.com%2Fecrd%2F2020%2FPosters%2FTheme%25201%2FP012_Siglec-6_a_potential_new_biomarker_for_clonal_mast_cell_diseases.pdf&usg=AOvVaw0n42dssQWsFsQ4fN3h-TsG.

[95] Human Proteinatlas. https://www.proteinatlas.org/ENSG00000105492-SIGLEC6.

[96] Bakker A.B.H., Oudenrijn S.V.D., Bakker A.Q., Feller N., Van Meijer M., Bia J.A., Jongeneelen M.A.C., Visser T.J., Bijl N., Geuijen C.A.W. (2004). C-Type Lectin-Like Molecule-1. Cancer Res..

[97] Ma H., Padmanabhan I.S., Parmar S., Gong Y. (2019). Targeting CLL-1 for acute myeloid leukemia therapy. J. Hematol. Oncol..

[98] Kenderian S.S., Ruella M., Shestova O., Klichinsky M., Kim M., Soderquist C., Bagg A., Singh R., Richardson C., Young R. (2017). Targeting CLEC12A with Chimeric Antigen Receptor T Cells Can Overcome the Chemotherapy Refractoriness of Leukemia Stem Cells. Biol. Blood Marrow Transplant..

[99] Zhang H., Wang P., Li Z., He Y., Gan W., Jiang H. (2021). Anti-CLL1 Chimeric Antigen Receptor T-Cell Therapy in Children with Relapsed/Refractory Acute Myeloid Leukemia. Clin. Cancer Res..

[100] Gomes-Silva D., Atilla E., Atilla P.A., Mo F., Tashiro H., Srinivasan M., Lulla P., Rouce R.H., Cabral J.M., Ramos C.A. (2019). CD7 CAR T Cells for the Therapy of Acute Myeloid Leukemia. Mol. Ther..

[101] Lynn R.C., Feng Y., Schutsky K., Poussin M., Kalota A., Dimitrov D.S., Powell Jr D.J. (2016). High-affinity FRβ-specific CAR T cells eradicate AML and normal myeloid lineage without HSC toxicity. Leukemia.

[102] Danylesko I., Jacoby E., Yerushalmi R., Shem-Tov N., Besser M.J., Vernitsky H., Marcu-Malina V., Shimoni A., Avigdor A., Nagler A. (2020). Remission of acute myeloid leukemia with t(8;21) following CD19 CAR T-cells. Leukemia.

[103] Gurney M., Stikvoort A., Nolan E., Kirkham-McCarthy L., Khoruzhenko S., Shivakumar R., Zweegman S., Van de Donk N.W., Mutis T., Szegezdi E. (2020). CD38 knockout natural killer cells expressing an affinity optimized CD38 chimeric antigen receptor successfully target acute myeloid leukemia with reduced effector cell fratricide. Haematologica.

[104] Baumeister S.H., Murad J., Werner L., Daley H., Trebeden-Negre H., Gicobi J.K., Schmucker A., Reder J., Sentman C.L., Gilham D.E. (2018). Phase I Trial of Autologous CAR T Cells Targeting NKG2D Ligands in Patients with AML/MDS and Multiple Myeloma. Cancer Immunol. Res..

[105] Hasegawa A., Saito S., Narimatsu S., Nakano S., Nagai M., Ohnota H., Inada Y., Morokawa H., Nakashima I., Morita D. (2021). Mutated GM-CSF-based CAR-T cells targeting CD116/CD131 complexes exhibit enhanced anti-tumor effects against acute myeloid leukaemia. Clin. Transl. Immunol..

[106] He X., Feng Z., Ma J., Ling S., Cao Y., Gurung B., Wu Y., Katona B.W., O’Dwyer K.P., Siegel D.L. (2020). Bispecific and split CAR T cells targeting CD13 and TIM3 eradicate acute myeloid leukemia. Blood.

[107] Chen N., Xu Y., Mou J., Rao Q., Xing H., Tian Z., Tang K., Wang M., Wang J. (2021). Targeting of IL-10R on acute myeloid leukemia blasts with chimeric antigen receptor-expressing T cells. Blood Cancer J..

[108] Le Q., Castro S., Tang T., Loeb A.M., Hylkema T., McKay C.N., Perkins L., Srivastava S., Call L., Smith J.L. (2021). Therapeutic Targeting of Mesothelin with Chimeric Antigen Receptor T Cells in Acute Myeloid Leukemia. Clin. Cancer Res..

[109] Lichtman E.I., Du H., Shou P., Song F., Suzuki K., Ahn S., Li G., Ferrone S., Su L., Savoldo B. (2021). Preclinical Evaluation of B7-H3–specific Chimeric Antigen Receptor T Cells for the Treatment of Acute Myeloid Leukemia. Clin. Cancer Res..

[110] Johnson L.A., Morgan R.A., Dudley M.E., Cassard L., Yang J.C., Hughes M.S., Kammula U.S., Royal R.E., Sherry R.M., Wunderlich J.R. (2009). Gene therapy with human and mouse T-cell receptors mediates cancer regression and targets normal tissues expressing cognate antigen. Blood.

[111] Abbott R.C., Cross R.S., Jenkins M.R. (2020). Finding the Keys to the CAR: Identifying Novel Target Antigens for T Cell Redirection Immunotherapies. Int. J. Mol. Sci..

[112] The Cancer Genome Atlas Research Network (2013). Genomic and epigenomic landscapes of adult de novo acute myeloid leukemia. N. Engl. J. Med..

[113] Van Der Lee D.I., Reijmers R.M., Honders M.W., Hagedoorn R.S., De Jong R.C., Kester M.G., Van Der Steen D.M., De Ru A.H., Kweekel C., Bijen H.M. (2019). Mutated nucleophosmin 1 as immunotherapy target in acute myeloid leukemia. J. Clin. Investig..

[114] Schumacher T., Bunse L., Pusch S., Sahm F., Wiestler B., Quandt J., Menn O., Osswald M., Oezen I., Ott M. (2014). A vaccine targeting mutant IDH1 induces antitumour immunity. Nature.

[115] Gambacorti-Passerini C., Grignani F., Arienti F., Pandolfi P.P., Pelicci P.G., Parmiani G. (1993). Human CD4 lymphocytes specifically recognize a peptide representing the fusion region of the hybrid protein pml/RAR alpha present in acute promyelocytic leukemia cells. Blood.

[116] Xie G., Ivica N.A., Jia B., Li Y., Dong H., Liang Y., Brown D., Romee R., Chen J. (2020). CAR-T cells targeting a nucleophosmin neoepitope exhibit potent specific activity in mouse models of acute myeloid leukaemia. Nat. Biomed. Eng..

[117] Rafiq S., Purdon T.J., Daniyan A.F., Koneru M., Dao T., Liu C., A Scheinberg D., Brentjens R.J. (2016). Optimized T-cell receptor-mimic chimeric antigen receptor T cells directed toward the intracellular Wilms Tumor 1 antigen. Leukemia.

[118] Masuda K., Hiraki A., Fujii N., Watanabe T., Tanaka M., Matsue K., Ogama Y., Ouchida M., Shimizu K., Ikeda K. (2007). Loss or down-regulation of HLA class I expression at the allelic level in freshly isolated leukemic blasts. Cancer Sci..

[119] Adamia S., Bar-Natan M., Haibe-Kains B., Pilarski P.M., Bach C., Pevzner S., Calimeri T., Avet-Loiseau H., Lode L., Verselis S. (2014). NOTCH2 and FLT3 gene mis-splicings are common events in patients with acute myeloid leukemia (AML): New potential targets in AML. Blood.

[120] Bordignon C., Bonini C., Verzeletti S., Nobili N., Maggioni D., Traversari C., Giavazzi R., Servida P., Zappone E., Benazzi E. (1995). Transfer of the HSV-tk Gene into Donor Peripheral Blood Lymphocytes for In Vivo Modulation of Donor Anti-Tumor Immunity after Allogeneic Bone Marrow Transplantation. The San Raffaele Hospital, Milan, Italy. Hum. Gene Ther..

[121] Bonini C., Bondanza A., Perna S.K., Kaneko S., Traversari C., Ciceri F., Bordignon C. (2007). The Suicide Gene Therapy Challenge: How to Improve a Successful Gene Therapy Approach. Mol. Ther..

[122] Straathof K.C., Pule M., Yotnda P., Dotti G., Vanin E.F., Brenner M.K., Heslop H.E., Spencer D.M., Rooney C.M. (2005). An inducible caspase 9 safety switch for T-cell therapy. Blood.

[123] Zhou X., Naik S., Dakhova O., Dotti G., Heslop H.E., Brenner M.K. (2016). Serial Activation of the Inducible Caspase 9 Safety Switch After Human Stem Cell Transplantation. Mol. Ther..

[124] Paszkiewicz P.J., Fräßle S.P., Srivastava S., Sommermeyer D., Hudecek M., Drexler I., Sadelain M., Liu L., Jensen M.C., Riddell S.R. (2016). Targeted antibody-mediated depletion of murine CD19 CAR T cells permanently reverses B cell aplasia. J. Clin. Investig..

[125] Loff S., Dietrich J., Meyer J.-E., Riewaldt J., Spehr J., von Bonin M., Gründer C., Swayampakula M., Franke K., Feldmann A. (2020). Rapidly Switchable Universal CAR-T Cells for Treatment of CD123-Positive Leukemia. Mol. Ther.-Oncolytics.

[126] Borot F., Wang H., Ma Y., Jafarov T., Raza A., Ali A.M., Mukherjee S. (2019). Gene-edited stem cells enable CD33-directed immune therapy for myeloid malignancies. Proc. Natl. Acad. Sci. USA.

[127] Humbert O., Laszlo G.S., Sichel S., Ironside C., Haworth K.G., Bates O.M., Beddoe M.E., Carrillo R.R., Kiem H.-P., Walter R.B. (2018). Engineering resistance to CD33-targeted immunotherapy in normal hematopoiesis by CRISPR/Cas9-deletion of CD33 exon 2. Leukemia.

[128] Mardiana S., Gill S. (2020). CAR T Cells for Acute Myeloid Leukemia: State of the Art and Future Directions. Front. Oncol..

[129] Majzner R.G., Mackall C.L. (2018). Tumor Antigen Escape from CAR T-cell Therapy. Cancer Discov..

[130] Liu F., Cao Y., Pinz K., Ma Y., Wada M., Chen K., Ma G., Shen J., Tse C.O., Su Y. (2018). First-in-Human CLL1-CD33 Compound CAR T Cell Therapy Induces Complete Remission in Patients with Refractory Acute Myeloid Leukemia: Update on Phase 1 Clinical Trial. Blood.

[131] Liu F., Zhang H., Sun L., Li Y., Zhang S., He G., Yi H., Wada M., Pinz K.G., Chen K.H. First-in-human cll1-cd33 compound car (ccar) t cell therapy in relapsed and refractory acute myeloid leukemia. Proceedings of the 25th EHA Annual Congress.

[132] Kloss C.C., Condomines M., Cartellieri M., Bachmann M., Sadelain M. (2012). Combinatorial antigen recognition with balanced signaling promotes selective tumor eradication by engineered T cells. Nat. Biotechnol..

[133] Cho J.H., Collins J.J., Wong W.W. (2018). Universal Chimeric Antigen Receptors for Multiplexed and Logical Control of T Cell Responses. Cell.

[134] Choe J.H., Watchmaker P.B., Simic M.S., Gilbert R.D., Li A.W., Krasnow N.A., Downey K.M., Yu W., Carrera D.A., Celli A. (2021). SynNotch-CAR T cells overcome challenges of specificity, heterogeneity, and persistence in treating glioblastoma. Sci. Transl. Med..

[135] Srivastava S., Salter A.I., Liggitt D., Yechan-Gunja S., Sarvothama M., Cooper K., Smythe K.S., Dudakov J.A., Pierce R.H., Rader C. (2019). Logic-Gated ROR1 Chimeric Antigen Receptor Expression Rescues T Cell-Mediated Toxicity to Normal Tissues and Enables Selective Tumor Targeting. Cancer Cell.

[136] Barrett A.J. (2019). Acute myeloid leukaemia and the immune system: Implications for immunotherapy. Br. J. Haematol..

[137] Hossain D.M.S., Dos Santos C., Zhang Q., Kozlowska A., Liu H., Gao C., Moreira D., Swiderski P., Jozwiak A., Kline J. (2014). Leukemia cell–targeted STAT3 silencing and TLR9 triggering generate systemic antitumor immunity. Blood.

[138] Mellor A.L., Keskin D.B., Johnson T., Chandler P., Munn D. (2002). Cells Expressing Indoleamine 2,3-Dioxygenase Inhibit T Cell Responses. J. Immunol..

[139] Xu H., Oriss T.B., Fei M., Henry A.C., Melgert B.N., Chen L., Mellor A.L., Munn D.H., Irvin C.G., Ray P. (2008). Indoleamine 2,3-dioxygenase in lung dendritic cells promotes Th2 responses and allergic inflammation. Proc. Natl. Acad. Sci. USA.

[140] Huang Q., Xia J., Wang L., Wang X., Ma X., Deng Q., Lü Y., Kumar M., Zhou Z., Li L. (2018). miR-153 suppresses IDO1 expression and enhances CAR T cell immunotherapy. J. Hematol. Oncol..

[141] Ninomiya S., Narala N., Huye L., Yagyu S., Savoldo B., Dotti G., Heslop H.E., Brenner M.K., Rooney C.M., Ramos C.A. (2015). Tumor indoleamine 2,3-dioxygenase (IDO) inhibits CD19-CAR T cells and is downregulated by lymphodepleting drugs. Blood.

[142] Frumento G., Rotondo R., Tonetti M., Damonte G., Benatti U., Ferrara G.B. (2002). Tryptophan-derived Catabolites Are Responsible for Inhibition of T and Natural Killer Cell Proliferation Induced by Indoleamine 2,3-Dioxygenase. J. Exp. Med..

[143] Atilla P.A., McKenna M.K., Tashiro H., Srinivasan M., Mo F., Watanabe N., Simons B.W., Stevens A.M., Redell M.S., E Heslop H. (2020). Modulating TNFα activity allows transgenic IL15-Expressing CLL-1 CAR T cells to safely eliminate acute myeloid leukemia. J. Immunother. Cancer.

[144] Batra S.A., Rathi P., Guo L., Courtney A.N., Fleurence J., Balzeau J., Shaik R.S., Nguyen T.P., Wu M.-F., Bulsara S. (2020). Glypican-3–Specific CAR T Cells Coexpressing IL15 and IL21 Have Superior Expansion and Antitumor Activity against Hepatocellular Carcinoma. Cancer Immunol. Res..

[145] Mathew N.R., Baumgartner F., Braun L., O’Sullivan D., Thomas S., Waterhouse M., Müller T., Hanke K., Taromi S., Apostolova P. (2018). Sorafenib promotes graft-versus-leukemia activity in mice and humans through IL-15 production in FLT3-ITD-mutant leukemia cells. Nat. Med..

[146] Chen X., Eksioglu E.A., Zhou J., Zhang L., Djeu J., Fortenbery N., Epling-Burnette P., Van Bijnen S., Dolstra H., Cannon J. (2013). Induction of myelodysplasia by myeloid-derived suppressor cells. J. Clin. Investig..

[147] Movahedi K., Guilliams M., Bossche J.V.D., Bergh R.V.D., Gysemans C., Beschin A., De Baetselier P., Van Ginderachter J. (2008). Identification of discrete tumor-induced myeloid-derived suppressor cell subpopulations with distinct T cell–suppressive activity. Blood.

[148] Mehta R.S., Chen X., Antony J., Szabolcs P. (2015). Myeloid Derived Suppressor Cells (MDSC)-like Acute Myeloid Leukemia (AML) Cells Are Associated with Resistance to Cytotoxic Effects of Autologous (Auto) T-Lymphocytes (CTLs). Biol. Blood Marrow Transplant..

[149] Epperly R., Gottschalk S., Velasquez M.P. (2020). A Bump in the Road: How the Hostile AML Microenvironment Affects CAR T Cell Therapy. Front. Oncol..

[150] Serafini P., Meckel K., Kelso M., Noonan K., Califano J., Koch W., Dolcetti L., Bronte V., Borrello I. (2006). Phosphodiesterase-5 inhibition augments endogenous antitumor immunity by reducing myeloid-derived suppressor cell function. J. Exp. Med..

[151] Orillion A., Hashimoto A., Damayanti N., Shen L., Adelaiye-Ogala R., Arisa S., Chintala S., Ordentlich P., Kao C., Elzey B. (2017). Entinostat Neutralizes Myeloid-Derived Suppressor Cells and Enhances the Antitumor Effect of PD-1 Inhibition in Murine Models of Lung and Renal Cell Carcinoma. Clin. Cancer Res..

[152] Mirza N., Fishman M., Fricke I., Dunn M., Neuger A.M., Frost T.J., Lush R.M., Antonia S., Gabrilovich D.I. (2006). All-trans-Retinoic Acid Improves Differentiation of Myeloid Cells and Immune Response in Cancer Patients. Cancer Res..

[153] Szczepanski M.J., Szajnik M., Czystowska-Kuźmicz M., Mandapathil M., Strauss L., Welsh A., Foon K.A., Whiteside T.L., Boyiadzis M. (2009). Increased Frequency and Suppression by Regulatory T Cells in Patients with Acute Myelogenous Leukemia. Clin. Cancer Res..

[154] Wan Y., Zhang C., Xu Y., Wang M., Rao Q., Xing H., Tian Z., Tang K., Mi Y., Wang Y. (2020). Hyperfunction of CD4 CD25 regulatory T cells in de novo acute myeloid leukemia. BMC Cancer.

[155] Hao K., Zhou Q., Chen W., Jia W., Zheng J., Kang J., Wang K., Duan T. (2012). Possible role of the ‘IDO-AhR axis’ in maternal-foetal tolerance. Cell Biol. Int..

[156] Curti A., Pandolfi S., Valzasina B., Aluigi M., Isidori A., Ferri E., Salvestrini V., Bonanno G., Rutella S., Durelli I. (2006). Modulation of tryptophan catabolism by human leukemic cells results in the conversion of CD25− into CD25+ T regulatory cells. Blood.

[157] Suryadevara C.M., Desai R., Farber S.H., Choi B.D., Swartz A.M., Shen S.H., Gedeon P.C., Snyder D.J., Herndon J.E., Healy P. (2018). Preventing Lck Activation in CAR T Cells Confers Treg Resistance but Requires 4-1BB Signaling for Them to Persist and Treat Solid Tumors in Nonlymphodepleted Hosts. Clin. Cancer Res..

[158] Schnorfeil F.M., Lichtenegger F.S., Emmerig K., Schlueter M., Neitz J.S., Draenert R., Hiddemann W., Subklewe M. (2015). T cells are functionally not impaired in AML: Increased PD-1 expression is only seen at time of relapse and correlates with a shift towards the memory T cell compartment. J. Hematol. Oncol..

[159] Chen X., Liu S., Wang L., Zhang W.-G., Ji Y., Ma X. (2008). Clinical significance of B7-H1(PD-L1)expression in human acute leukemia. Cancer Biol. Ther..

[160] Stahl M., Goldberg A.D. (2019). Immune Checkpoint Inhibitors in Acute Myeloid Leukemia: Novel Combinations and Therapeutic Targets. Curr. Oncol. Rep..

[161] Ding L., Ley T.J., Larson D., Miller C., Koboldt D.C., Welch J.S., Ritchey J.K., Young M.A., Lamprecht T.L., McLellan M.D. (2012). Clonal evolution in relapsed acute myeloid leukaemia revealed by whole-genome sequencing. Nature.

[162] Miles L.A., Bowman R.L., Merlinsky T.R., Csete I.S., Ooi A.T., Durruthy-Durruthy R., Bowman M., Famulare C., Patel M.A., Mendez P. (2020). Single-cell mutation analysis of clonal evolution in myeloid malignancies. Nature.

[163] Morita K., Wang F., Jahn K., Hu T., Tanaka T., Sasaki Y., Kuipers J., Loghavi S., Wang S.A., Yan Y. (2020). Clonal evolution of acute myeloid leukemia revealed by high-throughput single-cell genomics. Nat. Commun..

[164] Vasu S., Karl-Heinz H., Cheney C., Gopalakrishnan B., Mani R., Lozanski G., Mo X., Groh V., Whitman S.P., Konopitzky R. (2016). Decitabine enhances anti-CD33 monoclonal antibody BI 836858–mediated natural killer ADCC against AML blasts. Blood.

[165] You L., Han Q., Zhu L., Zhu Y., Bao C., Yang C., Lei W., Qian W. (2020). Decitabine-Mediated Epigenetic Reprograming Enhances Anti-leukemia Efficacy of CD123-Targeted Chimeric Antigen Receptor T-Cells. Front. Immunol..

[166] Xu N., Tse B., Yang L., Tang T.C., Haber M., Micklethwaite K., Dolnikov A. (2021). Priming Leukemia with 5-Azacytidine Enhances CAR T Cell Therapy. ImmunoTargets Ther..

[167] Wang Y., Tong C., Dai H., Wu Z., Han X., Guo Y., Chen D., Wei J., Ti D., Liu Z. (2021). Low-dose decitabine priming endows CAR T cells with enhanced and persistent antitumour potential via epigenetic reprogramming. Nat. Commun..

[168] Li S., Xue L., Wang M., Qiang P., Xu H., Zhang X., Kang W., You F., Xu H., Wang Y. (2019). Decitabine enhances cytotoxic effect of T cells with an anti-CD19 chimeric antigen receptor in treatment of lymphoma. OncoTargets Ther..

[169] Lee J.B., Khan D.H., Hurren R., Xu M., Na Y., Kang H., Mirali S., Wang X., Gronda M.V., Jitkova Y. (2021). Venetoclax enhances T cell-mediated anti-leukemic activity by increasing ROS production. Blood.

[170] Casares N., Pequignot M.O., Tesniere A., Ghiringhelli F., Roux S., Chaput N., Schmitt E., Hamai A., Hervas-Stubbs S., Obeid M. (2005). Caspase-dependent immunogenicity of doxorubicin-induced tumor cell death. J. Exp. Med..

[171] Vacchelli E., Ma Y., Baracco E.E., Sistigu A., Enot D.P., Pietrocola F., Yang H., Adjemian S., Chaba K., Semeraro M. (2015). Chemotherapy-induced antitumor immunity requires formyl peptide receptor 1. Science.

[172] Chulanetra M., Morchang A., Sayour E., Eldjerou L., Milner R., Lagmay J., Cascio M., Stover B., Slayton W., Chaicumpa W. (2020). GD2 chimeric antigen receptor modified T cells in synergy with sub-toxic level of doxorubicin targeting osteosarcomas. Am. J. Cancer Res..

[173] Ersvaer E., Brenner A.K., Vetås K., Reikvam H., Bruserud Ø. (2015). Effects of cytarabine on activation of human T cells—cytarabine has concentration-dependent effects that are modulated both by valproic acid and all-trans retinoic acid. BMC Pharmacol. Toxicol..

[174] Singh N., Perazzelli J., Grupp S.A., Barrett D.M. (2016). Early memory phenotypes drive T cell proliferation in patients with pediatric malignancies. Sci. Transl. Med..

[175] Tsuda K., Yamanaka K., Kitagawa H., Akeda T., Naka M., Niwa K., Nakanishi T., Kakeda M., Gabazza E., Mizutani H. (2012). Calcineurin Inhibitors Suppress Cytokine Production from Memory T Cells and Differentiation of Naïve T Cells into Cytokine-Producing Mature T Cells. PLoS ONE.

[176] He X., Smeets R.L., Koenen H.J.P.M., Vink P.M., Wagenaars J., Boots A.M.H., Joosten I. (2011). Mycophenolic Acid-Mediated Suppression of Human CD4+ T Cells: More Than Mere Guanine Nucleotide Deprivation. Arab. Archaeol. Epigr..

[177] Eyquem J., Mansilla-Soto J., Giavridis T., van der Stegen S.J.C., Hamieh M., Cunanan K.M., Odak A., Gönen M., Sadelain M. (2017). Targeting a CAR to the TRAC locus with CRISPR/Cas9 enhances tumour rejection. Nature.

[178] Poirot L., Philip B., Schiffer-Mannioui C., Le Clerre D., Chion-Sotinel I., Derniame S., Potrel P., Bas C., Lemaire L., Galetto R. (2015). Multiplex Genome-Edited T-cell Manufacturing Platform for “Off-the-Shelf” Adoptive T-cell Immunotherapies. Cancer Res..

[179] Sommer C., Cheng H.-Y., Nguyen D., Dettling D., Yeung Y.A., Sutton J., Hamze M., Valton J., Smith J., Djuretic I. (2020). Allogeneic FLT3 CAR T Cells with an Off-Switch Exhibit Potent Activity against AML and Can Be Depleted to Expedite Bone Marrow Recovery. Mol. Ther..

[180] Locke F.L., Malik S., Tees M.T., Neelapu S.S., Popplewell L., Abramson J.S., McDevitt J.T., Shin C.R., Demirhan E., Konto C. (2021). First-in-human data of ALLO-501A, an allogeneic chimeric antigen receptor (CAR) T-cell therapy and ALLO-647 in relapsed/refractory large B-cell lymphoma (R/R LBCL): ALPHA2 study. J. Clin. Oncol..

[181] Osborn M.J., Webber B.R., Knipping F., Lonetree C.-L., Tennis N., DeFeo A.P., McElroy A.N., Starker C., Lee C., Merkel S. (2016). Evaluation of TCR Gene Editing Achieved by TALENs, CRISPR/Cas9, and megaTAL Nucleases. Mol. Ther..

[182] Rozenbaum M., Meir A., Aharony Y., Itzhaki O., Schachter J., Bank I., Jacoby E., Besser M.J. (2020). Gamma-Delta CAR-T Cells Show CAR-Directed and Independent Activity Against Leukemia. Front. Immunol..

[183] Liu E., Marin D., Banerjee P., Macapinlac H.A., Thompson P., Basar R., Kerbauy L.N., Overman B., Thall P., Kaplan M. (2020). Use of CAR-Transduced Natural Killer Cells in CD19-Positive Lymphoid Tumors. N. Engl. J. Med..

[184] Bachanova V., Cayci Z., Lewis R.D., Maakaron J.E., Janakiram M., Bartz A., Payne B.S., Wong M.C., Cooley S., Valamehr B. (2020). Initial Clinical Activity of FT596, a First-in-Class, Multi-Antigen Targeted, Off-the-Shelf, iPSC-Derived CD19 CAR NK Cell Therapy in Relapsed/Refractory B-Cell Lymphoma. Blood.

